# Burnout Syndrome in Middle and Senior Management in the Industrial Manufacturing Sector of Mexico

**DOI:** 10.3390/ijerph16081467

**Published:** 2019-04-25

**Authors:** Sharon Macias-Velasquez, Yolanda Baez-Lopez, Aidé Aracely Maldonado-Macías, Jorge Limon-Romero, Diego Tlapa

**Affiliations:** 1 Facultad de Ingeniería, Arquitectura y Diseño, Universidad Autónoma de Baja California; Carretera Transpeninsular Ensenada-Tijuana 3917, Colonia Playitas, Ensenada 22860, Baja California, Mexico; idali.macias@uabc.edu.mx (S.M.-V.); jorge.limon@uabc.edu.mx (J.L.-R.); diegotlapa@uabc.edu.mx (D.T.); 2 Department of Industrial Engineering and Manufacturing, Autonomous University of Ciudad Juarez, Ave. del Charro 450 Norte, Cd Juarez 32310, Chihuahua, Mexico; amaldona@uacj.mx

**Keywords:** burnout, construct validity, MBI, industry

## Abstract

Due to globalization and the accelerated growth of technology, ever more employees of companies are affected by burnout syndrome, the psychological nature of which requires a prolonged response to chronic interpersonal stressors in work environments. The present research aims to validate the operability of the Maslach Burnout Inventory-General Survey (MBI-GS) using a sample of 378 professionals belonging to middle and senior management working in companies within the IMMEX sector (comprising the industrial-manufacturing, maquiladora and export services) located in the state of Baja California, Mexico. Firstly, an exploratory factor analysis using the principal components method and Varimax rotation was performed and the results revealed the existence of three factors representing more than 67 percent of the total variance. Secondly, a confirmatory factorial analysis was carried out performing appropriate results for the indices Chi-square goodness-of-fit model, Root Mean Square Error of Approximation (RMSEA), Normed Fit Index (NFI), Comparative Fit Index (CFI), Relative Fit Index (RFI), Parsimony Ratio (PRATIO) and Parsimony Normed Fit Index (PNFI), which are highly recommended by literature in these types of studies. Additionally, construct validity was satisfactorily verified. The factorial solution coincided with the Maslach Burnout Inventory original proposal so that this instrument can be considered a valid and reliable option to analyze the burnout levels in people pertaining to middle and senior management in these types of industries.

## 1. Introduction

Job stress is a collection of psychological, emotional, cognitive and behavioral reactions to certain overwhelming or demanding aspects of the organization and environment of the workplace. People presenting stress experience tension, anxiety and feelings of impotence at not being able to confront certain situations and, in some cases, repercussions for their physical health [1]. The seriousness of these symptoms will depend on the magnitude of the demands which the employee must fulfill in a certain period of time, their application of self-control and the decision-making freedom they enjoy [2]. Employees with demanding jobs characterized by work-based tension and low levels of decision-making autonomy and social support tend to take more sick-leave [3].

Job stress is among the main work-related problems according to the World Health Organization (WHO), and is a category in which Mexico is ranked first in the world, with 75% of its workers suffering from stress, followed by China and the United States, the two largest economies in the world, with 73% and 59%, respectively [4]. In economic terms, both workers and governments are noting ever higher costs of stress, which infringes on individual companies and the economy in general, affecting productivity [5] through the increased incidence of phenomena such as: working days lost due to absenteeism; internal employee transfers; rate of workplace accidents; client complaints; and, demand for the training of replacement workers [6]. This situation could represent losses of between 0.5% and 3.5% of a country’s gross domestic product [7].

In the particular case of Mexico, employee job stress causes losses of between 5 and 40 billion dollars per year [7]. Together with the existing difficulties in controlling other more well-known workplace risks, there is neither sufficient awareness of job stress nor sufficient resources for combatting it, with the concept being introduced into Mexican legislation little by little.

Job stress has been a growing subject of research, with the results obtained clearly showing the relationships among stress, health conditions [8,9,10], support from work colleagues [11] and behavior showing feelings of insecurity about work [10], to mention but a few areas. An extreme variant known as burnout appears in those individuals who are not able to successfully deal with extended periods of job stress [12,13]. From an existential perspective, the root cause of burnout resides in people’s need to believe that their lives are meaningful—that what they do is useful and important [14]. Those people hoping to obtain a meaning from their work develop their profession with highly-motivated and idealistic goals. When such people feel that they have failed, that their work is meaningless, and that they are not making a difference to the world, they begin to experience exhaustion and hopelessness, feelings which will, ultimately, consume them [15].

The concept of burnout was introduced by Maslach and Jackson and is the most widely used concept for this phenomenon in the literature [16]. Burnout began to appear with some regularity in the 1970s in the United States, especially among people working in the human services [17]. The conceptualization of job burnout is understood as a psychological syndrome in response to chronic interpersonal stressors on the job. The three key dimensions of this response are an overwhelming exhaustion, feelings of cynicism and detachment from the job, and a sense of ineffectiveness and lack of accomplishment [17].

The three dimensions of burnout are related to workplace variables in different ways. In general, exhaustion and cynicism tend to emerge from the presence of work overload and social conflict, whereas a sense of inefficacy arises more clearly from a lack of resources to get the job done (e.g., lack of critical information, lack of necessary tools, or insufficient time) [18]. In jobs with high job demands and limited job resources, we expect that employees develop exhaustion, cynicism, and a reduced sense of competence [19].

The main instrument used to measure burnout in a certain population is the Maslach Burnout Inventory-General Survey (MBI-GS), which was developed in response to two demands. The first comprised the study of the phenomenon in areas distinct to the healthcare sector for which it was originally created (MBI-HSS), finding, in some cases, different factorial structures with more than three dimensions, as in Densten [20]. Secondly, more studies have been born of an interest in the burnout caused by professional roles that do not necessarily involve the social demands of interacting with other individuals [21].

The validity of the instrument has been confirmed by various authors. Langballe [22] examined the factorial validity of the MBI-GS across eight different occupational groups in Norway: lawyers, physicians, nurses, teachers, church ministers, bus drivers, and people working in the advertising and information technology industries (*N* = 5024). Separate confirmatory factor analyses showed that the hypothesized three-factor model had sufficient fit in all occupational groups except for the group of people working in advertising. The results support that MBI-GS provides a suitable measurement to assess burnout across a diversity of professions [22]. Huibers et al. [23] carried out a cross-sectional study that compared the MBI-GS with other concepts such as chronic fatigue, finding that fatigued employees shared important characteristics with those suffering from chronic fatigue, independent of their state of burnout.

In the industrial sector, the validity of the Japanese translation of the MBI-GS was studied in a sample of middle management in a manufacturing company, with the results replicating the tri-dimensional structure of the original instrument and supporting the validity of the construct in the sample studied [24]. These studies confirmed factorial validity in the majority of the professions analyzed, maintaining the three-factor structure with which the concept was originally proposed.

Interest in this concept has increased in the last decade, both nationally and internationally [25], with the research panorama related to burnout in Latin America showing a tendency to follow the paradigmatic and methodological guidelines for studies carried out in the United States, Spain and other countries. This is reflected in the fact that the majority of the studies apply the Maslach and Jackson model. With regard to the professions of interest, a tendency to focus on the fields of healthcare and care provision was found, as well as those fields in which a supplier-client interaction can be clearly observed [26].

In Mexico, after nearly 20 years since the first publication on burnout, an increasing amount of research into this area [25] has focused on different professional populations, with teaching [27,28,29,30] healthcare [31,32] and high performance athletics [33] among those professions that stand out. The cities in which the highest number of studies on this subject have been conducted are Guadalajara and Mexico City [25].

This study was carried out in the main cities of the state of Baja California due to the great importance it represents to the industrial sector, occupying the first place in the northwest of the country in terms of the total number of people with employment, with a 56.4% participation rate [34].

In Baja California the activity of the maquiladoras began with the establishment of the free trade area in the 1960s, which allowed the duty-free importation of machinery, equipment, raw materials and supplies, after which the maquiladoras were extended to the rest of the region from the northern border, later, to the coasts, other border regions and, finally, to the whole country [35]. According to the data reported about the export manufacturing industry, in 2018, the industrial-manufacturing, maquiladora and export services (IMMEX) experienced a period of growth, with an increase of 0.51% in the number of companies [36]. The state of Baja California occupies the first place in the number of IMMEX companies, with 18.18%, and the second national place of personnel employed in the sector, with 14.64% [37]. In addition, 15,353 million dollars were generated in Baja California as export value, highlighting the importance of this activity in regional economy [35]. Due to the importance of this sector, the personnel of the IMMEX sector were selected to carry out the study, which noted the complexity of the functions they perform and the few studies that have been done on the burnout occurring in the sector, despite the growing industrial activity in the state.

Specifically, those positions at the middle and senior management were analyzed. Related research can be found among other studies conducted on this classification of personnel, such as the analysis of burnout and obesity [38] or burnout and the body weight index, which describe how important it is to take into account additional factors such as genetics or dietary habits [39]. In contrast to previous research, the present study proposes the validity of the instrument MBI-GS for a specific sector, geographic area and professional activity.

### The Present Research

Is necessary to broaden the occupational field and regions in which studies on burnout are carried out, improve the research design and information analysis, and ensure the validity of the scales used [25]. The present research aims to validate the operability of the MBI-GS for measuring the burnout construct in a sample of 378 professionals pertaining to middle and senior management in the industrial sector of the state of Baja California, Mexico, whose particular characteristics qualify them for evaluation in the selected sample.

Further to the evaluation of functionality being a fundamental requirement for every instrument used, the present study took into account the fact that the instrument has been translated into Spanish and that its design did not consider aspects of Latin American culture. The study was undertaken in order to validate the burnout construct with the objective of confirming that the instrument is being used correctly, independent of the subject’s position in the company, and their gender, age and/or profession [21,40], as other authors have confirmed in their studies.

The study begins by developing an exploratory factorial analysis (EFA) in order to determine the coincidence, in the sample selected, of the tridimensionality of the instrument.

Subsequently, confirmatory factorial analysis (CFA) is conducted with the objective of corroborating that the factorial structure is appropriate for use with the population of middle and senior management in the border region of the country.

Considering that the instrument can be used in any employment context, it is hoped that the application of the instrument in this sector obtains reliable measure of burnout’s levels. Specifically, the following hypotheses are tested:

**Hypothesis** **1:** *The tridimensional character of the MBI-GS is maintained for the population of middle and senior management in the industrial sector of the state of Baja California, Mexico.*

**Hypothesis** **2:** *The MBI-GS is a valid and reliable instrument for the measurement of burnout in middle and senior management in the state of Baja California, Mexico.*

## 2. Materials and Methods

### 2.1. Instrument Overview

The instrument used to measure burnout in the sample was the Spanish version of the MBI-GS, translated by Moreno et al. [41]. It is the generic version of the MBI Human Services Survey (HSS) used for professions outside the area of health, and comprises 16 items with 7 Likert-scale answer options for each question, where: 0 = on no occasion over the course of the year; 1 = very rarely over the course of the year; 2 = on some occasions over the course of the year; 3 = on many occasions over the course of the year; 4 = frequently over the course of the year; 5 = almost every day; and, 6 = every day. Maslach and Leiter describe the instrument’s 3 dimensions: Exhaustion (Exha) is the central quality of burnout and the most obvious manifestation of this complex syndrome and refers to feelings of being overextended and depleted of one’s emotional and physical resources [17]. Which could, for example, be exemplified by the questionnaire question “I feel exhausted by the end of my working day”.

Cynicism (Cyn) is an attempt to put distance between oneself and service recipients by actively ignoring the qualities that make them unique and engaging people. Their demands are more manageable when they are considered impersonal objects of one’s work. It refers to a negative, callous, or excessively detached response to various aspects of the job [17] and could be exemplified by the sentence “I only want to do my job and not be bothered”.

The component of reduced efficacy (ProEf) or accomplishment represents the self-evaluation dimension of burnout. It refers to feelings of incompetence and a lack of achievement and productivity at work [17]. Could be represented by the question “In my opinion, I am very good at my job”.

Of the 16 items, 10 were characterized negatively and 6 positively, with Table 1 showing the feelings to which each item refers and each identification code used in the research.

The questionnaire is structured with an introduction, followed by the employee’s informed consent and the section of MBI-GS questions, and finishes with the person’s demographic data, which include the following: type of management position; size of the company; time in the current position; the respondent’s department; type of contract; hours worked per week; academic qualifications; marital status; and, gender.

The research conforms to the provisions of the Declaration of Helsinki in 1995 (as revised in Edinburgh 2000) [42], and all ethical guidelines were followed as required for conducting human research, including adherence to the legal requirements of Mexico.

The database was analyzed using the software IBM^®^ SPSS^®^ Statistics version 23 (IBM company, Chicago, IL, USA), 64 bits edition, with the Amos™ (analysis of moment structures) complementary package [43].

### 2.2. Methodology

The stages which comprise the methodology developed in the present study are described below.

#### 2.2.1. Sample Selection

The professionals who belong to the middle and senior management of the 930 IMMEX manufacturing companies in the state of Baja California are the subjects of study for this investigation. Not knowing exactly the number of people in these positions, the study population is considered as being unknown. To extract the sample from the population, we used the non-probabilistic method of sampling by quota [44] with two strata, the first case of middle management, including department supervisors, group leaders, engineers and professionals, while the second stratum includes Senior management, including area managers and plant managers.

Contact with the companies to publicize the project was made by telephone and by email with the human resources department. The criterion used to determine the number of individuals to survey for each quota was for the convenience of participation; for the stratum of middle managers, 338 surveys were considered, while for the stratum of top managers there were 40 surveys, integrating a total sample of 378 employees from the state of Baja California, Mexico.

Regarding the sample size required to obtain reliable conclusions from the factorial analysis, we surpass by far what is recommended as a general rule, that indicates that the minimum is to have at least five times as many observations as the number of variables to be analyzed, and the more acceptable sample size would have a 10:1 ratio [45]. Therefore, considering a total of 16 items in the questionnaire, a sample size of 160 responses should be gathered.

#### 2.2.2. Data Analysis

Due to the nature of the participants and settings where they were contacted, we decided to review all collected data during and after collection, similarly to reference [46]. Accordingly, we didn’t have missing data in the responses to the MBI-GS questionnaire owing to the fact, that in the event of a missing response, the interviewer asked if it was possible to complete the question with the person concerned. The detection of outliers in the sample was carried out by means of the Mahalanobis d-squared distance, a statistical measurement that represents the distance, in units of standard deviation, of a point relative to the centroid [47].

EFA was used to identify the number and composition of the factors set out in Hypothesis 1. In addition, CFA was used to verify the number of underlying dimensions of the instrument and the pattern of item relationships (factor loadings). CFA also assists in the determination of how a test should be scored [48].

With the objective of determining the pertinence of developing a factorial analysis from a statistical perspective, the adequacy of implementing factorial analysis in the sample was evaluated using the determinant of the correlation matrix; the Bartlett sphericity test; the Kaiser-Meyer-Olkin (KMO) index; and, the measure of sample adequacy (MSA) for each item.

The extraction of factors was achieved via the use of principal component. In order to facilitate interpretation, the initial matrix is rotated by means of the Varimax orthogonal method, which despite not being the simplest analytical solution of the existing methods, does show the clearest separation of the factors [45] and, being independent of the distributional assumptions, is less likely to produce inadequate solutions [49]. Those factors with an eigenvalue greater than 1 were selected for extraction. Subsequently, a CFA was developed with the objective of maintaining the groupings and ensuring that the model shows an adequate fit. The estimation used for the model was undertaken by means of maximum likelihood.

The model fit for the sample was evaluated by means of 7 fit indices: Chi-square (*χ*^2^); Root Mean Square Error of Approximation (RMSEA); Normed Fit Index (NFI); Comparative Fit Index (CFI); Relative Fit Index (RFI); Parsimony Ratio (PRATIO); and, Parsimony Normed Fit Index (PNFI).

## 3. Results

### 3.1. Assessment of Normality

The normality of each of the variables that comprise the instrument was evaluated by means of the standardized kurtosis index and, taking into account the absolute values obtained, shows that all are below the value of 3 recommended by DeCarlos [50]. The positive kurtosis values are found to be within the range of (0.194, 2.740), while the negative values are found to be within the range of (−0.843, −0.216); therefore, it is possible that the condition of univariate normality is fulfilled for the sample.

Evidence of extreme kurtosis with absolute values higher than 8 [51] is always a cause for concern, given that it is known to be exceptionally prejudicial in analysis used for structural equation modeling.

A prerequisite to verifying that multivariable normality has been fulfilled is to have fulfilled the condition of univariate normality in each observable variable [50]. The evaluation of this type of normality considers the normalized value of multivariate Mardia kurtosis, with the procedure carried out by comparing the Mardia coefficient for the data under study with a calculated value based on the formula p(p+2), where p is the number of variables observed in the model [52]. In this case, the assumption is verified by contrasting the multivariable kurtosis value obtained via the AMOS program with the calculations made using the formula proposed [51]. Considering 16 variables for the instrument, the calculation undertaken with the formula proposed gives a value of 288, while the multivariable kurtosis index was 74.921, which is lower than the value calculated with the formula of reference, enabling the assumption, in this case, of the multivariable normality hypothesis in the data set.

The presence of multicollinearity in the data, the occurrence of which would indicate that distinct variables measure the same or are closely related, was then reviewed. In this sense, the variance inflation factor (VIF) verifies that, if this index is greater than 10, the variable could be redundant [51], while the results for the indices indicate a maximum value of 5.793; therefore, it is possible to conclude that the multicollinearity problem is not present in this data set.

From the database, 25 responses were identified as outliers and eliminated, leaving a total of 378 valid responses from further analysis. This test considered a conservative level of statistical significance with a value of *p* < 0.001 [50].

### 3.2. Characteristics of the Sample

The representative sample comprised 378 employees, aged from 25 to 60 (Mean ± SD: 34.01 ± 9.41), of whom 36.7% were women, 63.3% were male and 92.2% had an indefinite contract, 5.7% of which were temporary workers and 2% were not specified. With regard to working hours per week, 55.5% of subjects reported spending approximately 48 h in the office, while 22.4% reported a total of 45 h, 11.4% reported 56 or more hours, 6.5% reported 42 h, and, finally, 4.1% reported spending a minimum of 32 h working. Of those interviewed, 91.8% of employees pertain to middle management and 5.7% to senior management. 6.5% of this population has been educated up to secondary level and 20.4% up to high school level, while more than half of the sample (67.3%) has an undergraduate degree and only 5.3% has a postgraduate degree.

### 3.3. Factor Analysis

#### 3.3.1. Exploratory Factor Analysis

The data were analyzed using an EFA to prove whether the structure of the instrument was similar to that established in Maslach’s initial proposal, as well as to identify the existence of items with non significant factorial loadings, which, in some studies, have been eliminated.

Previously to run the EFA, the aforementioned tests were conducted with the sample data, obtaining satisfactory results in all cases. Firstly, the determinant = 3.325 × 10^−5^ for the correlation matrix was statistically different to zero this result, indicating that there are variables with very high intercorrelations, for which reason the analysis is feasible. Later Bartlett’s test of sphericity which is based on testing the null hypothesis that the correlation matrix of measure variables, provided an identity matrix and resulted in an approximate value of *χ*^2^ = 3823.886, with 120 degrees of freedom and the *p* = 0.000. In this case, the null hypothesis is rejected, indicating that the correlation matrix is statistically different to the identity matrix. Finally, the Kaiser-Mayer-Olkin (KMO) test is used to measure the sampling adequacy, and a value of 0.885 was obtained which considered an adequate relationship among the variables [53]. In terms of the measurement of the sampling adequacy (MSA), in which the results for each variable in the anti-image correlation matrix are examined, all exceeded the threshold value of 0.5 [45], which indicates that the reduction of the variables in the present research is adequate. The satisfactory results obtained in this stage allow us to continue with the analysis by indicating that this database is suitable to perform the factor analysis.

The next stage involved the group of the 16 variables into distinct factors that will now represent the original data, with these combinations of the original variables denominated here as dimensions. The extraction of factors obtained values of 6.293 for the first dimension, 3.228 for the second and 1.219 for the last, which, together, represent 67.128% of the total explained variance. Due to the coincidences found with the grouping of the items in the present sample, the resulting dimensions are named according to the original scale [17] as emotional exhaustion (Exha), professional efficiency (ProEf) and cynicism (Cyn). Thus, we found support for Hypothesis 1.

Table 2 presents the results for the significant loadings for each factor on the instrument (16 items), with 0.4 the minimum value considered for determining the influence of the item on the factor relative to the sample size [45]. The grouping of the variables in the factors was clear in terms of the loading values, for which reason no ambiguity was found in determining the factor to which they would pertain.

According to the results, the Exha factor is characterized by the following items: 1—totally exhausted; 2—exhausted; 3—fatigued; 4—stressed; and, 6—exhausted1. The ProEf factor has high factorial loads for the items: 5—resolve; 7—contribution; 10—good; 11—carried out; 12—value; and, 16—efficacy. Finally, the following items comprise the Cyn factor: 8—interest; 9—enthusiasm; 13—bother; 14—indifferent; and, 15—doubt.

Once the significant loading had been identified, the analysis of the communalities presented in Table 2, which is the extent to which an item correlates with all other items; represents the quantity of explained variance for the factorial model in terms of each variable.

In this case, it was considered that at least half of the explained variance in each variable was obtained, as suggested by Joseph Hair [45]. The portions of the explained variance with the greatest representativeness on the instrument were items 1, 2, 4, 6, 8, 9 and 14, with values above 0.7. It was observed that the acceptable levels of explanation were fulfilled for items 3, 7, 10, 11, 12, 15 and 16, while, finally, two items (5 and 13) were identified with values below established levels, indicating the lack of sufficient explanation for the factorial model analyzed. This stage was finalized, taking into account all the items comprising the instrument for subsequent analysis.

#### 3.3.2. Confirmatory Factorial Analysis

The most direct method for validating the results is to use a confirmatory perspective and evaluate the replicability of the results [45] in the sample of middle and senior management in the maquiladora industry of the state of Baja California. The importance of evaluating the quality of the results lies in ensuring the validity of the conclusions obtained [45]. For this, an objective evaluation was developed by means of a CFA complemented by a literature review that confirmed the grouping of the variables in the dimensions proposed, as well as a prior CFA. In particular, this section is developed with the aim of testing the degree to which the theoretical pattern of factor loading in specific dimensions represents the real data taken from the sample.

**Figure 1:**
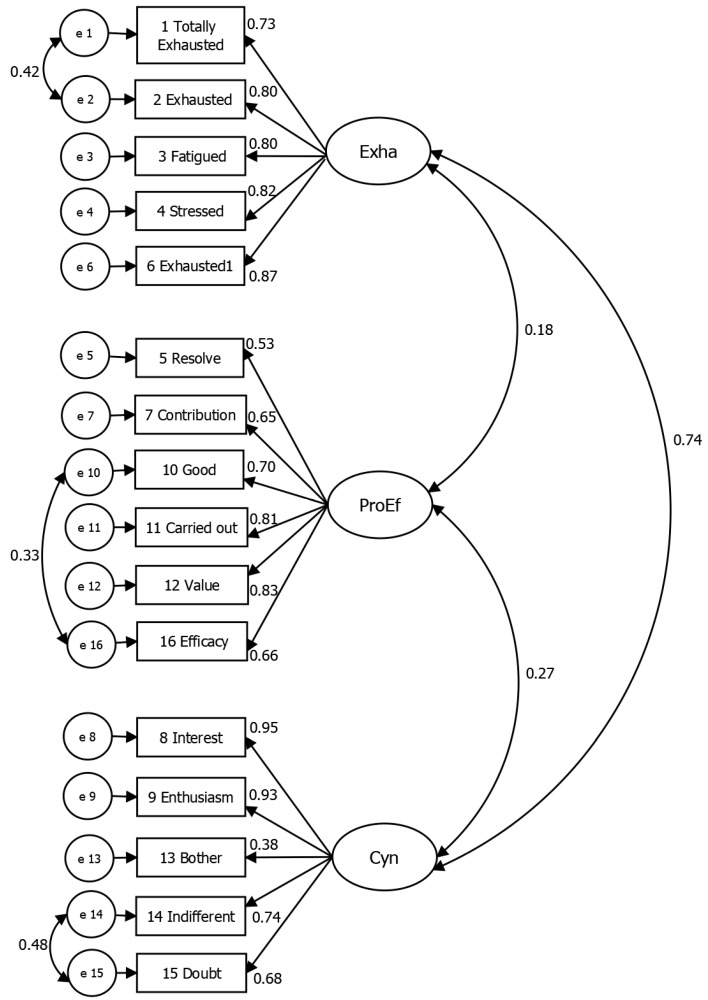
Model of factorial structure for MBI-GS in of middle and senior management in the maquiladora industry of the state of Baja California.

Figure 1 presents a measurement model that represents the same factorial structure on which the theoretical relationship proposed by Maslach [17] is based, as constituted by 3 latent correlational dimensions: Exha, with 5 variables and 5 associated errors (*e*); ProEf, with 6 variables and 6 associated errors (*e*); and, finally, Cyn, with 5 variables and 5 associated errors (*e*). These errors refer to inaccuracies in measuring the true variable values associated to every item in the questionnaire.

The comparison of the results of the testing of the theoretical measurement model with the reality represented in the sample is examined below, along with an analysis of both the global fit of the model and the validity criteria of the construct.

The global fit of the model is then analyzed, with Table 3 showing the final statistics produced by the CFA. *χ²* represents a traditional value for evaluating the fit of the model. A good fit should show non-significant results with a threshold of 0.05 [54]. The results show an *χ²* of 262.915 with a significant *p*-value; however, despite its popularity, there are various considerations that limit its use, known by some authors as the “lack of fit” [55] of a measurement, for which reason, it is recommended that it is accompanied by other fit statistics.

The value of the RMSEA index was 0.067, which is lower than 0.08 [56] for the model, with 16 variables measured in a sample of 378 data points. Using 90% of the highly significant confidence interval and a p-value for test of close fit (PCLOSE) of 0.003 for RMSEA gives the conclusion that the true value is found to be between 0.057 and 0.077, which provides additional support for the model fit. The second index considered is the minimum discrepancy (CMIN) in the *χ²* value divided by its degrees of freedom (*df*), giving a value of 2.683, which is notably lower than 3 [57]. In terms of the incremental fit, the CFI is the most widely used index, which, for this study, has a value of 0.956, which exceeds the threshold value of 0.95 [58].

The NFI index has a value greater than 0.9, with a good fit considered in accordance with Bentler and Bonnet [59], as does the RFI index. The parsimony indices consider the complexity of the model, which results in values considerably lower than other goodness of fit indices [60]. Mulaik et al. [55] state that it is possible to obtain parsimony of fit indices within the region 0.50–1.

The CFA results, with three relationships among the covariances of the error terms, suggest that the MBI measurement model gives a reasonably good fit, which supports continuing with the subsequent evaluations. Considering that one of the primary objectives of the CFA is to evaluate the validity of the construct of a theoretical measurement [45] this paper continues with the subsequent components that include said validity.

### 3.4. Convergent Validity

#### 3.4.1. Average Variance Extracted (AVE)

The items pertaining to a specific dimension must either converge in or share a high proportion of the common variance, which is known as convergent validity.

The convergent validity was obtained by means of the AVE, calculated as the average of the variance extracted from the standardized loading [45] of the items for each dimension expressed in the following manner (1):

(1) $$AVE=\frac{\sum_{i\;=\;1}^{n}L_{i}^{2}}{n}$$

where $L_{i}^{2}$ represents the loadings for the factor and *i* is the item number. For *n* items, the AVE is calculated as the total of the squared loadings divided by the number of items that comprise each factor. Values greater than 0.5 indicate an adequate convergence, with Table 4 showing values greater than the threshold for the Exha and Cyn dimensions, adequately fulfilling this principle, with the value for the ProEf dimension found to be very close to the established value.

#### 3.4.2. Reliability

It was necessary to determine whether the questions from the scale are related to each other. The Cronbach alpha index was used to measure the reliability of the internal consistency of the instrument [61]. This index oscillates between 0 and 1, where the closer the index is to 1, the greater the internal consistency of the items analyzed. For reference, values greater than 0.7 are considered acceptable, with results lower than this value considered questionable [62]. A value of 0.882 is obtained for the total instrument, while eliminating Item 13 gives the highest total value for the MBI-GS, 0.891, which coincides with the results of the confirmatory analysis.

The scores obtained for the dimensions for emotional exhaustion give a value of 0.908, the highest value obtained without deleting any of the items, while a value of 0.855 is obtained for professional efficiency without eliminating any item. Finally, it is suggested that, for cynicism, Item 13 is again eliminated, leading to the alpha value rising from 0.841 to 0.907. As shown in Table 4, despite the suggested elimination of an item from the Cyn dimension, the α values surpass 0.8, giving good internal consistency in the items comprising each dimension.

### 3.5. Discriminant Validity

This type of validity shows whether the dimensions forming the general concept of MBI are truly different to each other or whether it would be possible to group all the items in one single construct for the measurement model. This value is established by means of the AVE test and should be greater when compared with the estimated correlations for the other dimensions. Table 5 shows that the AVE values found on the diagonal are higher than the estimated correlations; therefore, for this example, the discriminant validity shows evidence that each construct is unique and can be used to analyze part of the phenomenon.

The results shown in the confirmatory factor analysis, as well as the tests of convergent and discriminant validity, confirm that the MBI-GS is a valid and reliable instrument for measuring burnout in middle and senior management in the state of Baja California, Mexico, as predicted by hypothesis 2.

## 4. Discussion

Taking into account the various studies that have been developed around the subject of chronic job stress, the main objective of the present research is to evaluate the operability of the MBI instrument by means of factorial validation analysis.

Further to being the most commonly used instrument [26] in the literature for detecting the degrees of burnout, one of the main advantages of the MBI is its validity, with high significant correlations found for the distinct sectors where it has been applied, in the analysis of professions providing social assistance, such as nurses [63], police officers [64], and teachers [65], or even in the comparison of employees by gender [66].

The results of the present study reveal this to be the most adequate instrument for evaluating burnout syndrome in middle and senior management [67]. Taking a maximum of approximately 5 min to complete, it is, therefore, viable for application in companies where there is limited time for activities of this nature (the application of questionnaires, guided tours, etc.); moreover, it is designed for upper and senior management, who are responsible for formulating, articulating and executing strategy and policy within their organizations and who often work more than 48 h per week.

As a limitation to this type of study, we have the fact that the participation of workers is voluntary, so the study can discard the opinion of people who do not agree to participate and there is a risk of bias in the information. However, for this particular study, the strategy was to present the project to senior management, through the Human Resources department, explaining the benefits of the same for the workers and convincing them of their participation, so there was no resistance to providing answers.

From the initial statistical analysis stage, the data in the model showed the existence of three correlated dimensions (Exha, ProEf and Cyn) on which the construct can be formulated. The factorial loading for the items obtained higher values than those previously established and enabled the easy identification as to which dimension they pertain. The tests undertaken to verify the adequacy of model were completed satisfactorily. For verifying the amount of explained variance via commonality analysis, 2 items were detected, items 5 and 13, with low values compared to the established threshold. The strategy for these two largely unrepresentative items was to conserve them in the model, firstly because all the conditions were correctly fulfilled in prior analysis, and secondly because the values are slightly lower than 0.5 and, in accordance with Hair [44] are not considered low commonalities. However, the possibility of eliminating them at some point was not discounted.

A CFA was developed to provide a confirmatory focus for the established theoretical measure and, in contrast with the EFA, the number of factors were specified *a priori*, as well as the number of variables loaded on these factors. From a confirmatory perspective, the fit for the model suggests the inclusion in the evaluation of at least one absolute index of fit and an incremental index of fit, in addition to Hair’s [45] *χ²* results; however, the present study evaluated 7 indices, obtaining satisfactory results for each. The Chi-square (*χ²*) test, the RMSEA and its respective confidence interval were considered for the absolute fit, while, for the incremental fit, the NFI, CFI and RFI were considered, and, finally, PRATIO and PNFI were evaluated for the parsimony fit.

The modification index (MI) analysis suggested the following correlations for residual errors, with values above 27.59 for items 14–15, 1–2 and 10–16 for cynicism, emotional exhaustion and professional efficiency, respectively. This correlation represented improvements in model fit with direct evidence in the indices described above, whose final values are those presented. Studies have been found which confirm the three-factor original model that enables correlation among residual errors, showing a better fit for healthcare professionals [21], lawyers, priests, teachers and drivers [22].

The extracted variance test was carried out to complement the analysis of the validity of the construct. While analyzing the estimations of the standardized weight applied to the AVE calculation, values ranging from 0.535 to 0.949 were observed. However, a value lower than 0.384 was obtained in the factor for Item 13 on the scale for cynicism, a result which coincided with the findings of Bria et al. [21], who reported a value of 0.21 for the same items, the lowest obtained in their research, which was, however, conducted with healthcare professionals and, thus, is distinct to the profession featured in the present study.

The Cronbach alpha method was used to evaluate internal consistency for each dimension of the instrument, whose values suggest that it is robust, obtaining α_Exha_ = 0.90, α_ProEf_ = 0.85 and α_Cyn_ = 0.84. These results were compared with averaged values from 7 studies [25], obtaining α_Exha_ = 0.86, α_ProEf_ = 0.75 and α_Cyn_ = 0.60, from which it can be clearly observed that, in all dimensions, the values are lower than the results, showing a better level of consistency for the sample data.

## 5. Conclusions

The importance of a having a valid instrument in place at the beginning of any project lies in the reliability of the results obtained in the final stages. For this reason, this study centered on investigating the grouping of the factors that have an impact on the burnout measured with the MBI-GS, which is considered the predominant instrument on an international level, used in up to 90% of studies [68]. It comprises 16 items mainly related to the individual-work interaction, leaving the feelings generated by workplace relations in second place, for example whether the people with whom the subject interacts could be the cause of burnout [69].

The results of the theoretically-founded factorial statistical analysis conducted in the study confirm the original three-factor structure proposed, with 6 items obtained for professional efficiency, 5 for emotional exhaustion, and 5 for cynicism.

The Hypothesis 1 was accepted because the exploratory analysis confirmed the factorial structure of the instrument, with this result confirmed in the subsequent stage.

The Hypothesis 2 was accepted based on the fulfillment of not only the indices of fit for the model, but also the convergent and discriminant validity and reliability on which the construct’s validity is based. The instrument has been shown to be adequate for detecting burnout syndrome in middle and senior management in the state of Baja California, Mexico.

The timely detection of burnout syndrome by companies will have repercussions in terms of the work climate and productivity, and also for the personal fulfillment of the employee.

In the international context, on World Day for Safety and Health at Work in 2013, the International Labor Organization (ILO) stated that, despite the fact that some workplace risks have decreased, an increase in new work-related health conditions has been registered without the application of adequate prevention, protection and control measures. Ergonomic risk factors are included among these emerging workplace risks.

It is for the above reason that ergonomic risk factors demand a governmental commitment to strengthen occupational health and safety to ensure dignified or decent work, through policies, strategic lines of action and projects with a preventive focus, in order to guarantee healthy and safe working conditions. With the health effects of these factors on employees being well-known, it is of the utmost importance to attend to them.

## Figures and Tables

**Table 1:** Codes of the MBI-GS (Maslach Burnout Inventory-General Survey) instrument.

Item Code	Feeling That Represents
1 Totally exhausted	Exhausted emotionally
2 Exhausted	Finishing at the end of day
3 Fatigued	Fatigued at dawn
4 Stressed	Work is stressful
5 Resolve	Able to solve problems
6 Exhausted1	Exhausted by my work
7 Contribution	Make a contribution to work
8 Interest	Loss of interest
9 Enthusiasm	Loss of enthusiasm
10 Good	I’m good at doing my job
11 Carried out	I feel fulfilled
12 Value	Realized worthwhile things
13 Bothered	Do not bother me
14 Indifferent	I have become indifferent
15 Doubt	I doubt the value of my work
16 Efficacy	Effective in doing my job

Based on Moreno [41].

**Table 2:** Matrix of rotated component y communalities.

Item Code	Factors			Communalities
	Exha	ProEf	Cyn	
1 Totally Exhausted	0.844			0.734
2 Exhausted	0.871			0.795
3 Fatigued	0.769			0.690
4 Stressed	0.786			0.717
6 Exhausted1	0.753			0.765
5 Resolve		0.658		0.441
7 Contribution		0.688		0.545
10 Good		0.814		0.678
11 Carried out		0.791		0.659
12 Value		0.830		0.694
16 Efficacy		0.763		0.606
8 Interest			0.768	0.786
9 Enthusiasm			0.740	0.761
13 Bother			0.652	0.442
14 Indifferent			0.774	0.748
15 Doubt			0.737	0.679
% Variance	39.334	20.173	7.621	
% Accumulated variance	39.334	59.507	67.128	

Note: Exha: emotional exhaustion; ProEf: professional efficiency; Cyn: cynicism.

**Table 3:** Summary of the adjustment indices of the measurement model.

Goodness of Fit Statistics
Chi-square (*χ*^2^) = 262.915 (*p* = 0.000)
Degrees of freedom (*df*) = 98 Absolute fit indices Root mean square error of approximation (RMSEA) = 0.067 90 percent confidence interval for RMSEA = (0.057; 0.077) Incremental fit indices
Normed fit index (NFI) = 0.932 Comparative fit index (CFI) = 0.956 Relative fit index (RFI) = 0.917 Parsimony fit indices
Parsimony Ratio (PRATIO) = 0.817
Parsimony normed fit index (PNFI) = 0.761

**Table 4:** Average Variance Extracted and α Cronbach by dimension.

Factors	Items	AVE	α Cronbach
Exha	1 Totally Exhausted	0.646	0.908
	2 Exhausted		
	3 Fatigued		
	4 Stressed		
	6 Exhausted1		
ProEf	5 Resolve	0.494	0.855
	7 Contribution		
	10 Good		
	11 Carried out		
	12 Value		
	16 Efficacy		
Cyn	8 Interest	0.585	0.841
	9 Enthusiasm		
	13 Bother		
	14 Indifferent		
	15 Doubt		

**Table 5:** Correlations among the dimensions, average variance extracted and squared correlations.

	Exha	ProEf	Cyn
Exha	0.804 ^a^		
ProEf	0.184	0.703 ^a^	
Cyn	0.742	0.268	0.765 ^a^

Note: ^a^ Root square of AVE.
